# Forecasting and change point test for nonlinear heteroscedastic time series based on support vector regression

**DOI:** 10.1371/journal.pone.0278816

**Published:** 2022-12-30

**Authors:** HsinKai Wang, Meihui Guo, Sangyeol Lee, Cheng-Han Chua

**Affiliations:** 1 Department of Applied Mathematics, National Sun Yat-sen University, Kaohsiung, Taiwan; 2 Department of Statistics, Seoul National University, Seoul, Korea; Institute for Economic Forecasting, Romanian Academy, ROMANIA

## Abstract

SVR-ARMA-GARCH models provide flexible model fitting and good predictive powers for nonlinear heteroscedastic time series datasets. In this study, we explore the change point detection problem in the SVR-ARMA-GARCH model using the residual-based CUSUM test. For this task, we propose an alternating recursive estimation (ARE) method to improve the estimation accuracy of residuals. Moreover, we suggest using a new testing method with a time-varying control limit that significantly improves the detection power of the CUSUM test. Our numerical analysis exhibits the merits of the proposed methods in SVR-ARMA-GARCH models. A real data example is also conducted using BDI data for illustration, which also confirms the validity of our methods.

## Introduction

Non-linearity and conditional heteroscedasticity are two major characteristics of financial time series data. The SVR-ARMA-GARCH models proposed by [1] provide flexible model fitting and good predictive ability for data with these two characteristics. However, major events such as the financial tsunami and the COVID-19 pandemic often bring shocks and changes to the financial market, making the original models no longer suitable for future decisions. Therefore, it is important to detect the occurrence of change points in time to update the model. The objective of this paper is twofold: the first is to improve the forecasting capability for more accurate parameter/residual estimation for the SVR-ARMA-GARCH time series, and the second is to enhance the detection power of the residual-based CUSUM test of [2] when changes occur near the current observing time.

For the first, we propose an alternating recursive estimation (ARE) method which estimates jointly the parameters of the conditional mean and conditional variance equations of SVR-ARMA-GARCH models. Our numerical studies show that the proposed ARE method outperforms [1] method in terms of forecasting performance and thereby improves the accuracy of parameter/residual estimation in SVR-ARMA-GARCH models. This improvement in the residual estimation also greatly enhances the detection ability of a change point.

For the second, we aim to improve the residual-based CUSUM test proposed by [2]. Their test only uses time series observations and model-based residuals, so that it has merits to be simpler and more flexible than other types of CUSUM tests. We refer to [3] for a theoretical background and history of CUSUM tests for time series, particularly, the ones based on residuals. Although [2] test performs adequately in many situations, *T*_max_ has a shortcoming to yield low powers when a change is near current observing time, which results in a late detection. To resolve this problem, we here propose an alternative testing procedure by supplying a time-varying upper control limit (UCL) for *T*_*s*_, wherein the underlying basic process asymptotically behaves like a standardized (by its time point) Brownian bridge, and additionally, the test statistic $T_{\text{max}}^{\text{scale}}$ of [4] whose limiting process is the supremum of a Brownian bridge standardized by its maximizing point.

Our numerical analysis reveals that those three tests have their own merits of yielding high powers in different stages of time period, namely, $T_{\text{max}}^{\text{scale}}$, *T*_max_, and *T*_*s*_ with UCL ($T_{s}^{\text{UCL}}$) perform the best for detecting a change in an early, middle and late stage, respectively. Since the CUSUM test under consideration is retrospective, the late stage of time period implicates the time close to the currently observing time, and therefore, the last testing procedure is conducive to quickly detecting a change point near the current time and makes a more suitable test for a dynamic monitoring scheme and timely update of models particularly when a moving window scheme is adopted.

The organization of this paper is as follows. Section 2 introduces the proposed alternating recursive estimation (ARE) method for the SVR-ARMA-GARCH model. Section 3 introduces the aforementioned three residual-based CUSUM tests. Simulation and empirical studies are conducted in Sections 4 and 5 for illustration. Section 6 provides the conclusions.

## Materials and methods

### ARE of the SVR-ARMA-GARCH models

Time series forecasting is crucial to forecast the characteristics of time series and detect anomalies in statistical monitoring process. Conventionally, linear ARMA models have been used for this purpose, but as time series often has significant nonlinear features, the forecasting result based on the ARMA models is inaccurate and hard to utilize for the application to monitoring processes. As an alternative, researchers can consider using support vector regression (SVR). SVR originates from [5, 6] statistical learning theory, which uses nonlinear functions to convert input variables into a higher dimensional space to helps explore information that can only be observed in higher dimensional space. Under the varieties of circumstances, it provides flexibility and excellent forecasting accuracy, and satisfies the structural risk minimization principle, see [7–9]. This far, the SVR has been applied to various practical problems; for example [10], used it for stock prediction and [11] used it for anomaly detection.

Chen et al. [1] adopted the SVR method to estimate the parameters in SVR-based nonlinear GARCH models and showed that the SVR-GARCH models significantly outperform classical parametric models in the respect of one-period-ahead volatility forecasting. In this study, we aim to improve and refine their method as addressed below.

Let *r*_*t*_ denote an asset return at time *t*. The *r*_*t*_ is said to follow an ARMA(*p*, *q*)-GARCH(*m*, *s*) model if

$$\begin{array}{cc} r_{t} & =\phi_{0}+\sum_{i=1}^{p}\phi_{i}r_{t-i}+\sum_{j=1}^{q}\theta_{j}a_{t-j}+a_{t} \end{array}$$ (1)

$$\begin{array}{cc} a_{t}^{2} & =\alpha_{0}+\sum_{i=1}^{\text{max}(m,s)}\left(\alpha_{i}+\beta_{i}\right)a_{t-i}^{2}-\sum_{j=1}^{s}\beta_{j}\eta_{t-j}+\eta_{t}, \end{array}$$ (2)

where $\eta_{t}=a_{t}^{2}-\sigma_{t}^{2}$ is a martingale difference sequence and $\sigma_{t}^{2}=\text{Var}\left(a_{t}|F_{t-1}\right).$ It is understood that *α*_*i*_ = 0 for *i* > *m* and *β*_*j*_ = 0 for *j* > *s*.

The SVR-ARMA-GARCH model replaces the linear functions in Eqs (1) and (2) with the nonlinear functions *h*(⋅) and *g*(⋅) and is expressed as:

$$\begin{array}{c} r_{t}=h\left(r_{t,p},a_{t,q}\right)+a_{t} \end{array}$$ (3)

$$\begin{array}{c} a_{t}^{2}=g\left(a_{t,\text{max}(m,s)}^{2},\eta_{t,s}\right)+\eta_{t}, \end{array}$$ (4)

where

$$\begin{array}{c} h\left(r_{t,p},a_{t,q}\right)=w_{h}^{T}\phi_{h}\left(r_{t,p},a_{t,q}\right), \end{array}$$

$$\begin{array}{c} g\left(a_{t,\text{max}(m,s)}^{2},\eta_{t,s}\right)=w_{g}^{T}\phi_{g}\left(a_{t,\text{max}(m,s)}^{2},\eta_{t,s}\right) \end{array}$$

with **r**_*t*,*p*_ = (*r*_*t*−1_, *r*_*t*−2_, …, *r*_*t*−*p*_) and **a**_*t*,*q*_ = (*a*_*t*−1_, *a*_*t*−2_, …, *a*_*t*−*q*_).

The functions *ϕ*_*h*_(⋅) and *ϕ*_*g*_(⋅) are defined using a Gaussian kernel, i.e.

$$\begin{array}{c} \phi_{h}^{T}\left(x\right)\phi_{h}\left(y\right)=\text{exp}\left(-\gamma_{h}∥x-y{∥}^{2}\right) \end{array}$$

$$\begin{array}{c} \phi_{g}^{T}\left(x\right)\phi_{g}\left(y\right)=\text{exp}\left(-\gamma_{g}∥x-y{∥}^{2}\right). \end{array}$$

Chen et al. [1] proposed to estimate **w**_*h*_ and **w**_*g*_ separately by optimizing the two objective functions in Eqs (5) and (6) below. Namely, Eq (5) is optimized to obtain **w**_*h*_ and the residual ${\hat{a}}_{t}=r_{t}-h\left(r_{t,p},{\hat{a}}_{t,q}\right)$, and ${\hat{a}}_{t}$ is used to optimize Eq (6) for obtaining **w**_*g*_:

$$\begin{array}{c} \min_{w_{h}}\frac{1}{2}∥w_{h}{∥}^{2}+C_{h}\sum_{t=1}^{n}\text{max}(|r_{t}-h\left(r_{t,p},{\hat{a}}_{t,q}\right)\left|-\epsilon_{h},0\right) \end{array}$$ (5)

and

$$\begin{array}{c} \min_{w_{g}}\frac{1}{2}∥w_{g}{∥}^{2}+C_{g}\sum_{t=1}^{n}\text{max}(|{\hat{a}}_{t}^{2}-g\left({\hat{a}}_{t,\text{max}(m,s)}^{2},{\hat{\eta }}_{t,s}\right)\left|-\epsilon_{g},0\right), \end{array}$$ (6)

However, this method does not consider the impact of volatility on the returns when estimating **w**_*h*_. As such, we replace the residuals in Eq (5) with the standardized residuals in Eq (7) to estimate **w**_*h*_ as follows:

$$\begin{array}{c} \min_{w_{h}}\frac{1}{2}{∥w_{h}∥}^{2}+C_{h}\sum_{t=1}^{n}\text{max}(\frac{|r_{t}-h\left(r_{t,p},{\hat{a}}_{t,q}\right)|-\epsilon_{h}}{{\hat{\sigma }}_{t}},0). \end{array}$$ (7)

**Fig 1:**
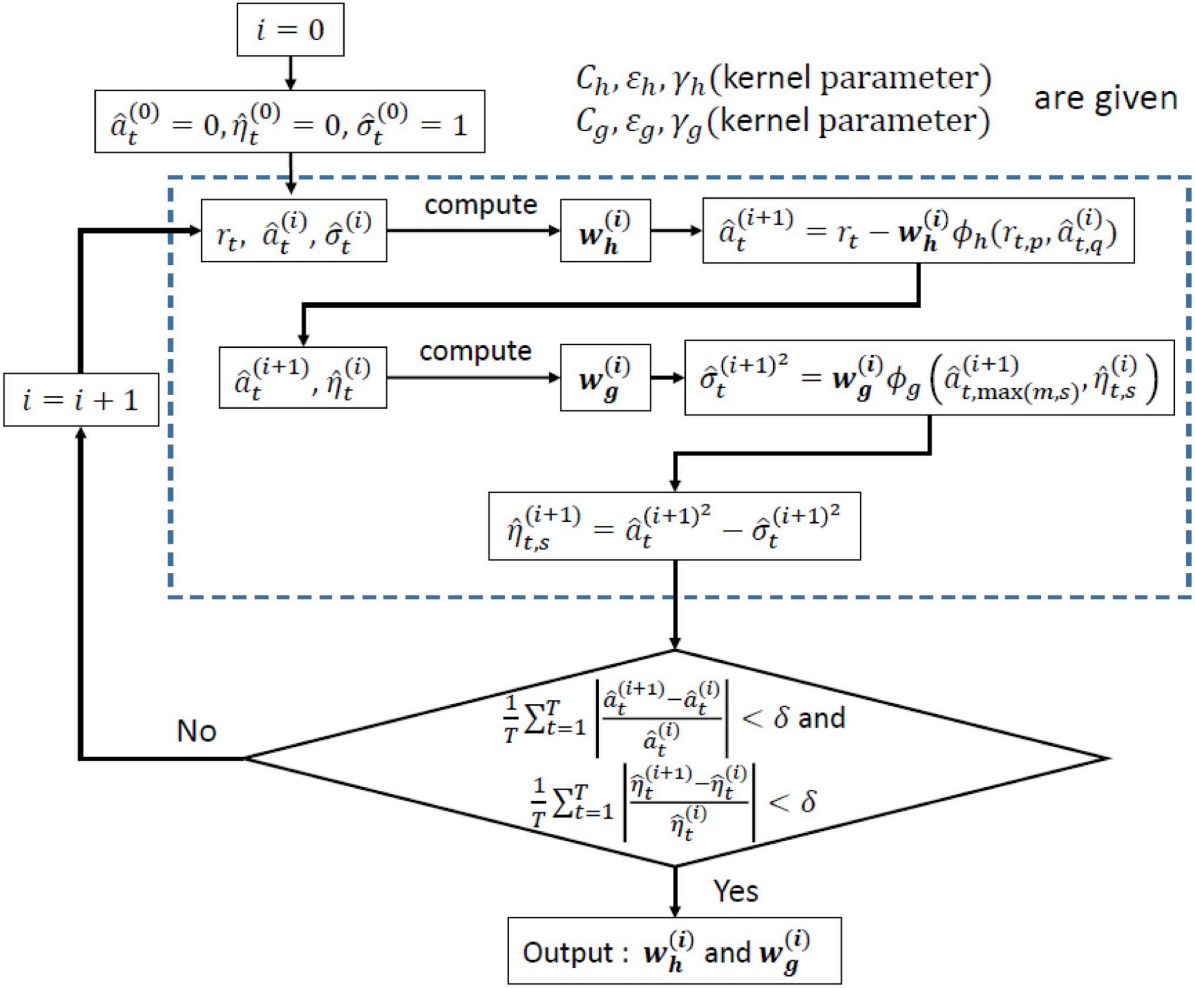
ARE of the SVR-ARMA-GARCH models.

To optimize Eqs (6) and (7), we adopt an alternating recursive estimation (ARE) method illustrated in Fig 1. Namely, at each iteration, we fix **w**_*g*_ to update **w**_*h*_, then fix **w**_*h*_ to update **w**_*g*_, and continue to alternate this estimation procedure until the two residual sequences sufficiently converge to each other. This method performs more functionally in computing the predicted values and residuals, used for detecting a change point as described in Section 4 below, wherein the predominance of the ARE method is demonstrated empirically through Monte Carlo simulations.

### Residual-based CUSUM test

This section introduces three different test statistics to detect a change point.

#### *T*_max_ of Lee et al. [2]

Let us consider the location-scale time series model of the form:

$$\begin{array}{c} y_{t}=h_{t}\left(y_{t-1},y_{t-2},...\right)+g_{t}^{1/2}\left(y_{t-1},y_{t-2},...\right)\eta_{t},\;\;t\in Z, \end{array}$$ (8)

where *η*_*t*_ are i.i.d. random variables with mean 0 and a fourth moment. The *h*_*t*_(⋅) and *g*_*t*_(⋅) indicate the conditional mean and variance of *y*_*t*_ at time *t*. A structural change occurs if *h*_*t*_(⋅) and *g*_*t*_(⋅) experiences a change. Given observations *y*_1_, …, *y*_*n*_, we set up the null and alternative hypotheses:

$$\begin{array}{c} H_{0}\;:\;h_{t}\left(\cdot \right)\;\text{and}\;g_{t}\left(\cdot \right)\;\text{remain}\;\text{the}\;\text{same}\;\text{for}\;\text{the}\;\text{whole}\;\text{period.}\;\;\text{vs}.\;\;H_{1}:\;\text{not}\;H_{0}. \end{array}$$

To test these hypotheses [2], used the test:

$$\begin{array}{c} T_{\text{max}}=\underset{s\in (0,1)}{\text{max}}\left(T_{s}\right) \end{array}$$

with

$$\begin{array}{c} T_{s}=\frac{1}{\sqrt{n}{\hat{\tau }}_{n}}|\sum_{i=1}^{\left[ns\right]}\left(y_{i}-{\hat{a}}_{i}\right){\hat{a}}_{i}-\left(\frac{\left[ns\right]}{n}\right)\sum_{i=1}^{n}\left(y_{i}-{\hat{a}}_{i}\right){\hat{a}}_{i}| \end{array}$$

where ${\hat{\tau }}_{n}^{2}=\frac{1}{n}\sum_{i=1}^{n}{\left(\left(y_{i}-{\hat{a}}_{i}\right){\hat{a}}_{i}\right)}^{2}$ and $\hat{a_{t}}=y_{t}-{\hat{h}}_{t}\left(\cdot \right)$ is the estimated residual at time *t*. Since the statistic *T*_*s*_ only involves observations and residuals, as far as the residuals of the model are estimable, the residual-based CUSUM test can be constructed for detecting a change point. For parametric location-scale models [3, 12], proved that under *H*_0_ and regularity conditions, *T*_max_ converges weakly to the sup of a standard Brownian bridge. Lee et al. [2] applied this paradigm to Model (8) with a hybrid of SVR methods and demonstrated its validity through empirical studies.

According to [13],

$$\begin{array}{c} P(\underset{s}{\text{sup}}|B_{s}\left|\leq x\right)=\frac{\sqrt{2\pi }}{x}\sum_{i=1}^{\infty }e^{-{\left(2i-1\right)}^{2}\pi^{2}/\left(8x^{2}\right)} \end{array}$$ (9)

Hence, for a given significance level *α*, we can find a critical value *K* from Eq (9) satisfying

$$\begin{array}{c} P(\text{sup}|B_{s}|>K)=\alpha . \end{array}$$ (10)

In particular, when *α* = 0.05, the corresponding *K* is 1.358. When *T*_max_ is greater than the critical value *K*, we reject the null hypothesis.

**Fig 2:**
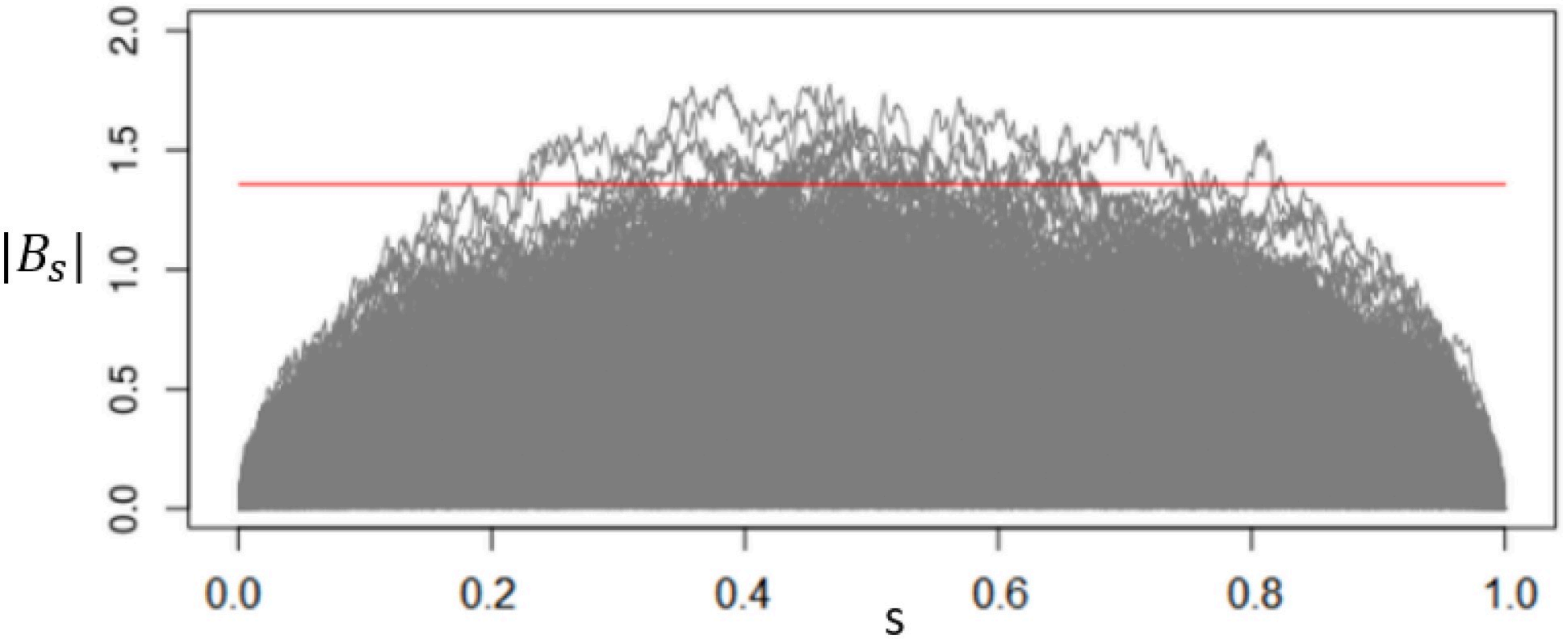
Control limit of the CUSUM test based on *T*_max_.

Fig 2 plots 500 paths of |*B*_*s*_|, where the red horizontal line corresponds to the critical value *K* = 1.358 for the significance level *α* = 0.05.

#### $T_{\text{max}}^{\text{scale}}$ of Ferger [4]

Instead of *T*_max_, one can consider another residual-based CUSUM test:

$$\begin{array}{c} T_{\text{max}}^{\text{scale}}=\frac{{\text{max}}_{s\in (0,1)}T_{s}}{\sqrt{s^{\prime }\left(1-s^{\prime }\right)}},\;\text{where}\;s^{\prime }=\text{arg}\underset{s\in (0,1)}{\text{max}}T_{s}, \end{array}$$ (11)

Ferger [4] obtained the limiting distribution of $T_{\text{max}}^{\text{scale}}$ and used it to improve the power of the Kolmogorov-Smirnov test. More specifically [4], proved that the c.d.f. of *R*:

$$\begin{array}{c} R=\frac{{\text{sup}}_{s\in (0,1)}|B_{s}|}{\sqrt{s^{\prime }\left(1-s^{\prime }\right)}},\;\text{where}\;s^{\prime }=\text{arg}\underset{s\in (0,1)}{\text{sup}}|B_{s}|, \end{array}$$ (12)

has a form of

$$\begin{array}{c} \begin{array}{cc} F_{R}\left(x\right)= & 16\sum_{0\leq j<l<\infty }{\left(-1\right)}^{j+l}\frac{\alpha_{j}\alpha_{l}}{\alpha_{l}^{2}-\alpha_{j}^{2}}(\frac{\Phi \left(\alpha_{j}x\right)-\frac{1}{2}}{\alpha_{j}}-\frac{\Phi \left(\alpha_{l}x\right)-\frac{1}{2}}{\alpha_{l}})+\\ & 4\sum_{0\leq j<\infty }(\frac{\Phi \left(\alpha_{j}x\right)-\frac{1}{2}}{\alpha_{j}}-x\phi \left(\alpha_{j}x\right)) \end{array} \end{array}$$ (13)

for *x* ≥ 0 and *α*_*i*_ = 2*i* + 1, where Φ and *ϕ* denote the distribution function and its density of a standard normal random variable. For a given significance level *α*, the null hypothesis is rejected if $T_{\text{max}}^{\text{scale}}$ is greater than the critical value $F_{R}^{-1}\left(\alpha \right)$. In particular, using Eq (13), we obtain $F_{R}^{-1}\left(0.05\right)=2.795$ and $F_{R}^{-1}\left(0.10\right)=2.5$.

#### *T*_*s*_ with a time-varying control limit

**Fig 3:**
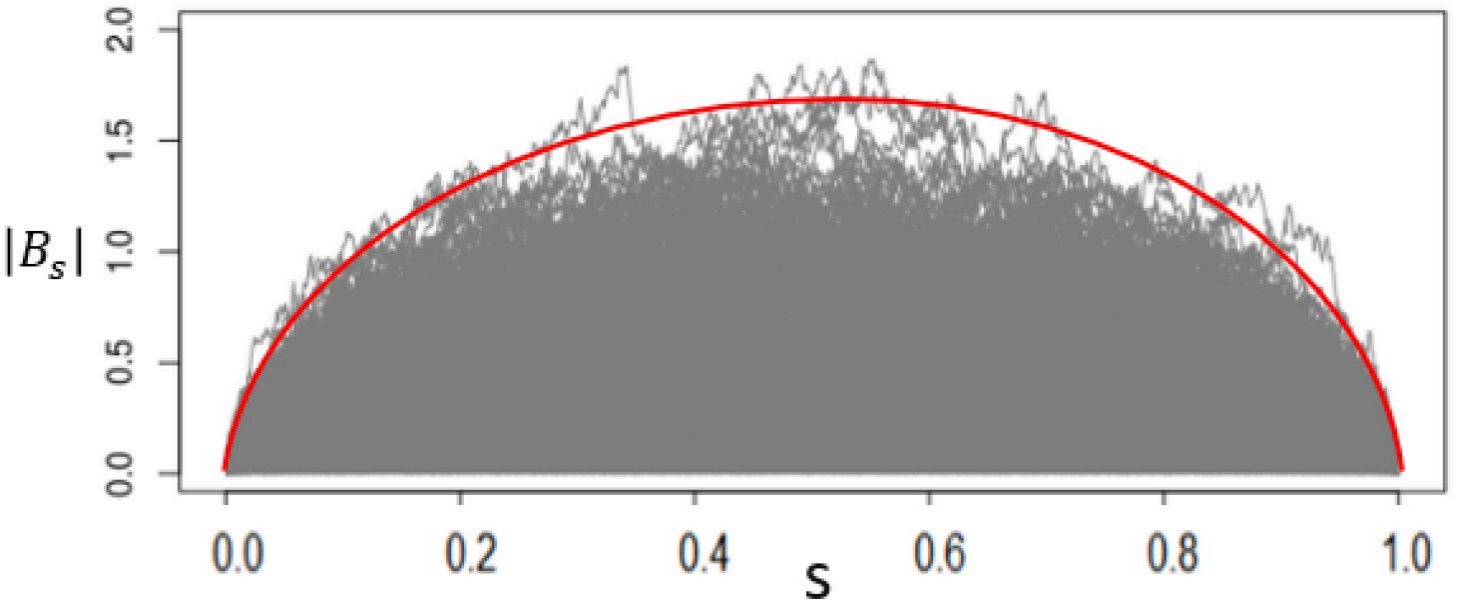
Control limit of |*B*_*s*_|.

Although $T_{\text{max}}^{\text{scale}}$ can overcome a shortcoming of *T*_max_, it only considers the standardized *T*_max_ but not the standardized values at other time points. As such, we here construct the time-varying control limits for *T*_*s*_ based on the fact:

$$\begin{array}{c} \lim_{n\to \infty }P(\underset{\frac{1}{n}\leq s\leq 1-\frac{1}{n}}{\text{sup}}(\frac{|B_{s}|}{\sqrt{s(1-s)}})\leq \frac{C+D_{n}}{A_{n}})=\text{exp}(-2e^{-C})=1-\alpha , \end{array}$$ (14)

where

$$\begin{array}{c} A_{n}=\sqrt{2\;\text{log}\;\text{log}\;n} \end{array}$$

and

$$\begin{array}{c} D_{n}=2\;\text{log}\;\text{log}n+\frac{1}{2}\text{log}\;\text{log}\;\text{log}n-\frac{1}{2}\text{log}\;\pi , \end{array}$$

refer to Corollary A.3.1 of [14]. For example, when *α* = 0.05 and *n* = 500, we have $\frac{C+D_{n}}{A_{n}}=3.536$. The time-varying control limit of *T*_*s*_ is exhibited in Fig 3.

This result enables us to set up an adjusted control limit for improvement, but for small *s*, there can be a concern regarding excessive type 1 errors in its implementation. For example, when the AR (1) model below considered,

$$\begin{array}{c} y_{t}=0.3y_{t-1}+a_{t}, \end{array}$$ (15)

where *a*_*t*_ are i.i.d. *N*(0, 1) errors, the test based on 500 observations from this model shows that the rejection ratio of the null hypothesis out of 500 replications is 0.114 which is much larger than the significance level of *α* = 0.05.

**Fig 4:**
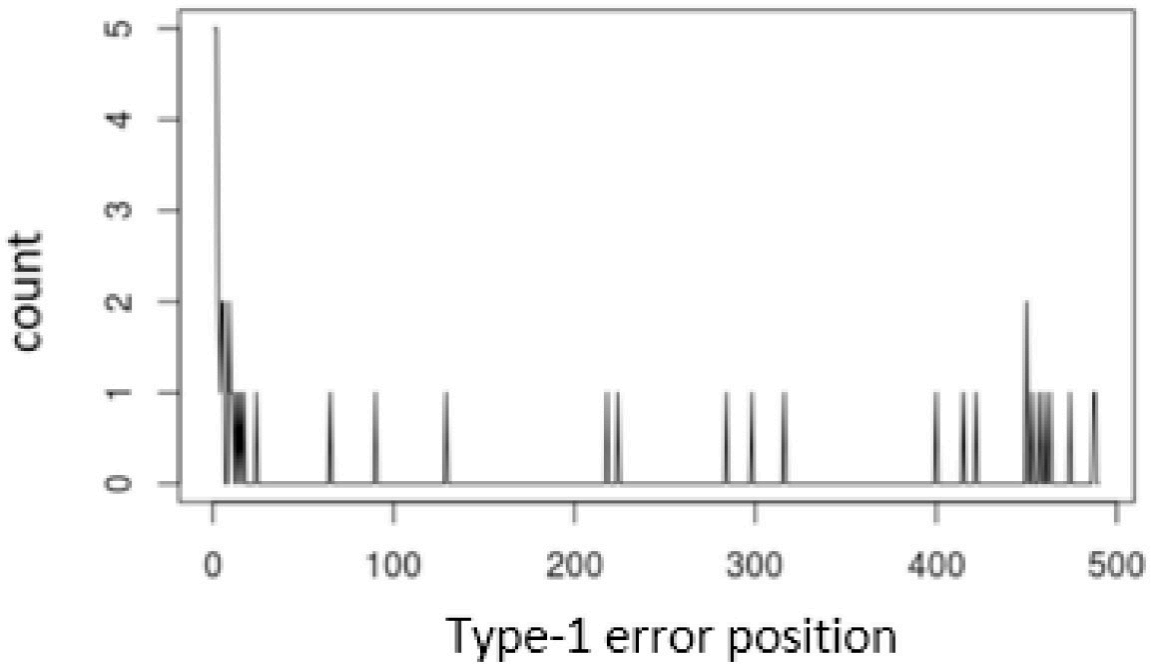
Type-1 error frequency of the AR(1) model based on Eq (14).

In Fig 4, the x-axis represents the time points where the type 1 error occurs, and the y-axis represents the number of occurrences. The figure indicates that the control limit based on Eq (14) cannot perform well in an early stage.

To overcome this problem, we propose to use the control limit for *T*_*s*_, *s* ∈ (0, 1), as follows:

$$\begin{array}{c} UCL\left(s\right)=\{\begin{array}{ccc} K & , & s<s^{*}\\ K^{*}\sqrt{s(1-s)} & , & s\geq s^{*} \end{array} \end{array}$$ (16)

Then, we reject the null hypothesis when *T*_*s*_ > *UCL*(*s*) for some *s* ∈ (0, 1). We name this testing method $T_{s}^{\text{UCL}}$.

For a given significance level *α*, the upper control limit can be obtained via finding *K*, K*, and s* in Eq (16) through the following steps:

1. Find *K* with *P*(sup|*B*_*s*_| > *K*) = *α*.
2. Simulate 100,000 standard Brownian bridge paths of sample size *n*.
3. Find K* ∈ [2, 4] with $s^{*}=\frac{1}{2}-\sqrt{\frac{1}{4}-\frac{K^{2}}{K^{*2}}}$ satisfying
  $$\begin{array}{c} \#(|B_{i}|<UCL\left(i\right),\forall i=\frac{\left\{1,2,...,n\right\}}{n+1})\approx 100,000\left(1-\alpha \right). \end{array}$$

**Fig 5:**
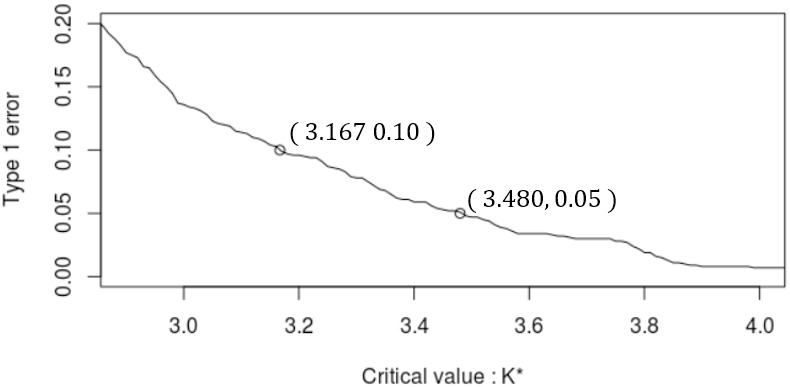
Type1 error probability *α* v.s. the critical value K*.

**Fig 6:**
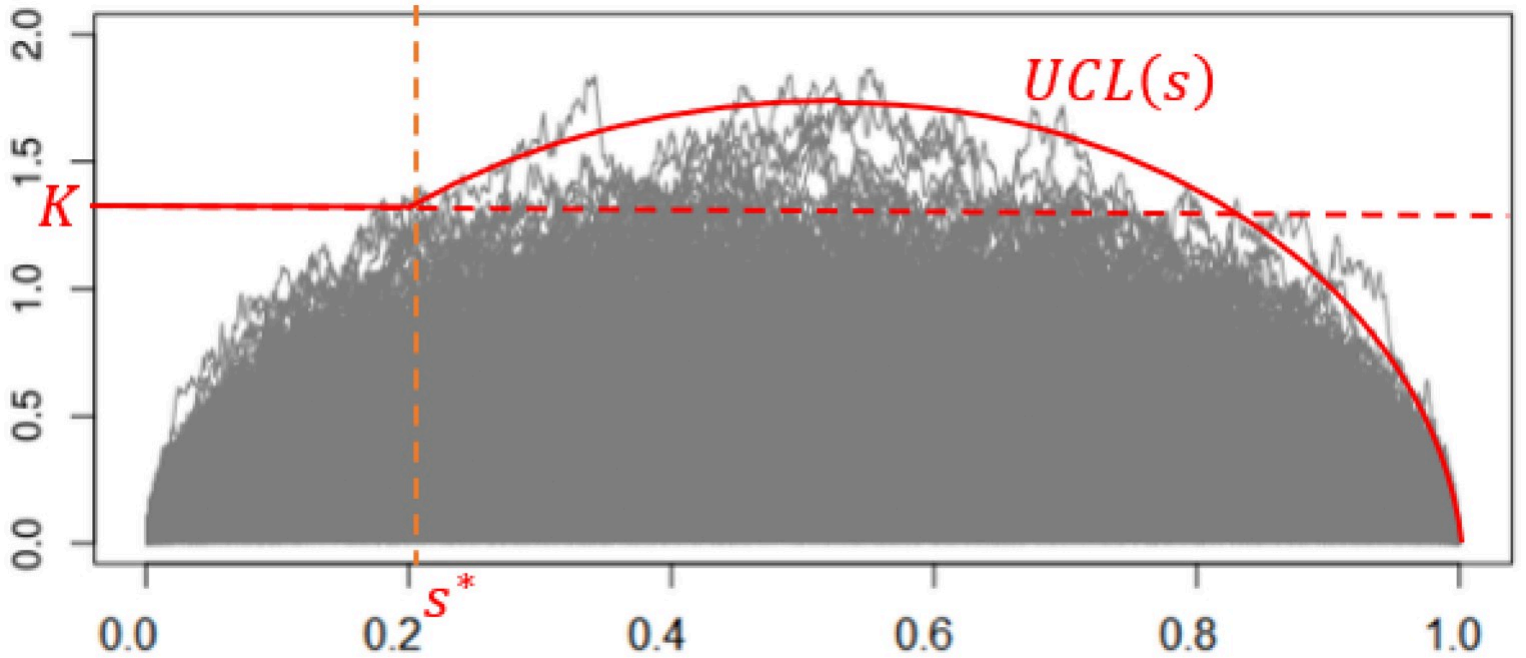
The upper control limit of *T*_*s*_ for *α* = 0.05.

Fig 5 shows the plot of *α* v.s. K* for *n* = 500. Fig 6 shows the upper control limit of *T*_*s*_ for *α* = 0.05 (the red solid line) which is determined by the following formula:

$$\begin{array}{c} UCL\left(s\right)=\{\begin{array}{ccc} 1.358 & , & s<0.206\\ 3.48\sqrt{s(1-s)} & , & s\geq 0.206 \end{array}. \end{array}$$ (17)

**Fig 7:**
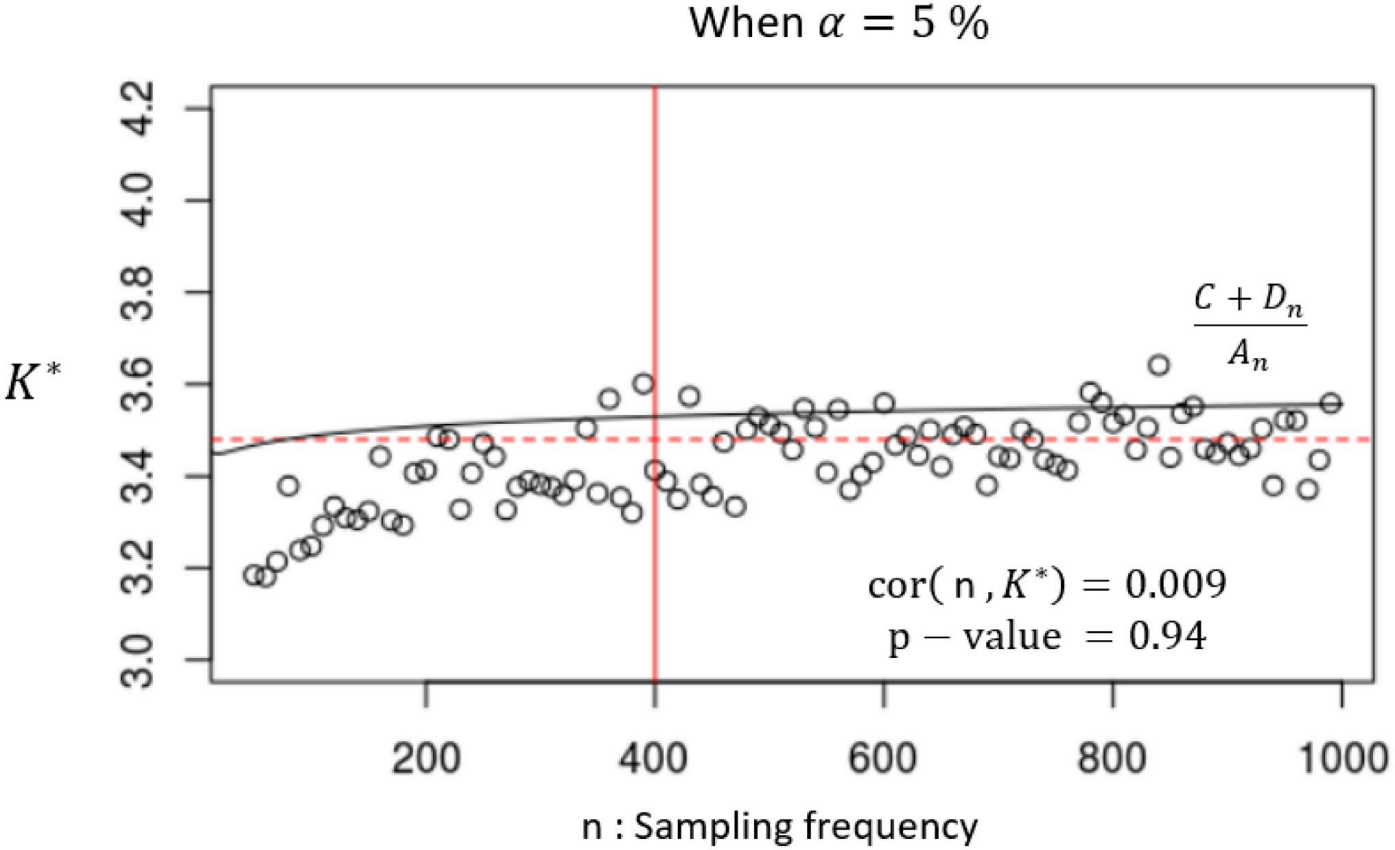
K* v.s. sample size *n*.

We further investigate the stability of the critical value K* vs. the sample size *n*. Fig 7 shows that when the sample size *n* < 400, the value of K* increases as *n* grows gradually, but when 400 ≤ *n* ≤ 1000, the value of K* looks stable.

## Results and discussion

### Numerical analysis

We evaluate the performance of the SVR-ARMA-GARCH model using the proposed ARE method and the residual-based CUSUM test based on *T*_*s*_. For this task, we generate the data following a Lorenz system, namely, *y*_*t*_, *t* = 1, 2, …, *n*, is from the following model:

$$\begin{array}{c} y_{t}=\phi \beta_{t}+\sqrt{|\gamma_{t}|}\eta_{t},\;\;\eta_{t}\;\underset{˜}{iid}\;N\left(0,1\right), \end{array}$$ (18)

where *β*_*t*_ and *γ*_*t*_ originate from the Lorenz system, as shown in Eq (19). Under the null of no changes, we consider *ϕ* = 1. The Lorentz system is a three-dimensional dynamic system, obtained from the convective volume equation in the atmospheric equation:

$$\begin{array}{c} \begin{array}{cc} \frac{\text{d}(\alpha_{t})}{\text{d}t} & =10(\beta_{t}-\alpha_{t})\\ \frac{\text{d}(\beta_{t})}{\text{d}t} & =\alpha_{t}\gamma_{t}+\frac{8}{3}\alpha_{t}-\beta_{t}\\ \frac{\text{d}(\gamma_{t})}{\text{d}t} & =\alpha_{t}\beta_{t}-28\gamma_{t}, \end{array} \end{array}$$ (19)

**Fig 8:**
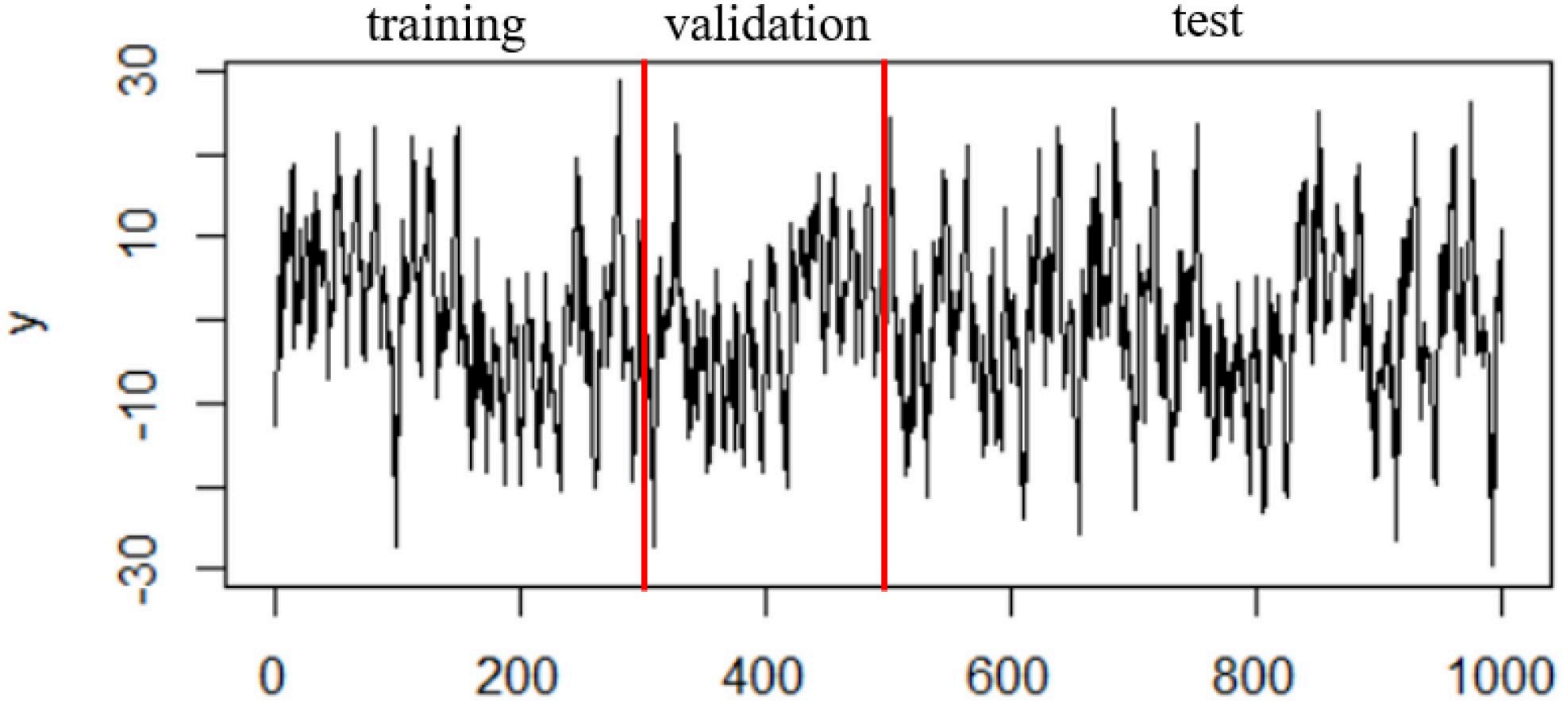
Time series simulated by Lorenz system.

For each simulation, 1100 observations are simulated, and the first 100 are removed. The first 500 observations are used as a training set to fit the three models: ARMA-GARCH, SVR-ARMA-GARCH based on the ARE and the method of [1], and the last 500 observations are used as a test set for prediction. The last 200 observations in the training set are used as a validation set to decide the free parameters of the SVR-ARMA-GARCH model. Fig 8 shows a time series realization simulated by Lorenz system and the partition of training, validation and testing sets. We use rolling window to evaluate the 500 one-step-ahead forecasts of the fitted three models respectively in the testing period.

#### Evaluation metrics

We use five evaluation metrics to measure the prediction accuracy. Let *y*_*t*_ denote the series of the returns to predict, and ${\hat{a}}_{t}=y_{t}-{\hat{y}}_{t}$ denote the estimated residuals.

- Evaluation metrics for conditional mean predictor ${\hat{y}}_{i}$
  (i) NMSE: Normalized mean square error based on *y*_*i*_,
    $$\begin{array}{c} \frac{\sum_{i=1}^{n}{\left(y_{i}-{\hat{y}}_{i}\right)}^{2}}{\sum_{i=1}^{n}{\left(y_{i}-{\bar{y}}_{i}\right)}^{2}}, \end{array}$$ (20)
  (ii) NMSE_COND: Normalized mean square error based on the conditional mean of *y*_*i*_,
    $$\begin{array}{c} \frac{\sum_{i=1}^{n}{\left(E\left(y_{i}|F_{i-1}\right)-{\hat{y}}_{i}\right)}^{2}}{\sum_{i=1}^{n}{\left(E\left(y_{i}|F_{i-1}\right)-E\left(y_{i}\right)\right)}^{2}}, \end{array}$$ (21)
  (iii) Sign error:
    $$\begin{array}{c} \frac{1}{|n^{\prime }|}\sum_{i\in n^{\prime }}I_{\left[y_{i}{\hat{y}}_{i}<0\right]},\;\text{where}\;n^{\prime }=\left\{t:{\hat{y}}_{t}>0.01\text{sd}\left({\hat{y}}_{t}\right)\right\}. \end{array}$$ (22)
- Evaluation metrics for conditional variance predictor ${\hat{\sigma }}_{i}^{2}$
  (i) RMSE_var: Root mean square error based on ${\hat{a}}_{i}^{2}$,
    $$\begin{array}{c} \sqrt{\sum_{i=1}^{n}\frac{{\left({\hat{\sigma }}_{i}^{2}-{\hat{a}}_{i}^{2}\right)}^{2}}{n}}, \end{array}$$ (23)
  (ii) RMSE_var_COND: Root mean square error based on conditional variance of *y*_*i*_,
    $$\begin{array}{c} \sqrt{\sum_{i=1}^{n}\frac{{\left({\hat{\sigma }}_{i}^{2}-\text{Var}\left(y_{i}|F_{i-1}\right)\right)}^{2}}{n}}. \end{array}$$ (24)

Table 1 shows the averaged performance for each evaluation metric after 500 repetitions. As the data is generated from the nonlinear Lorenz system, the performance of the ARMA-GARCH model is shown to be flawed. A pairwise t-test demonstrates that the ARE method performs significantly better than that of [1].

**Table 1:** Prediction comparison for model based on the Lorenz system.

	ARMA-GARCH	SVR-ARMA-GARCH	
		ARE	Chen *et al*. [1]
NMSE(%)	58.74	43.58	43.82
NMSE_COND(%)	31.24	20.36	20.67
Sign error(%)	30.32	23.84	24.04
RMSE_var(%)	79.58	67.23	67.72
RMSE_var_COND(%)	30.54	15.30	15.87

**Fig 9:**
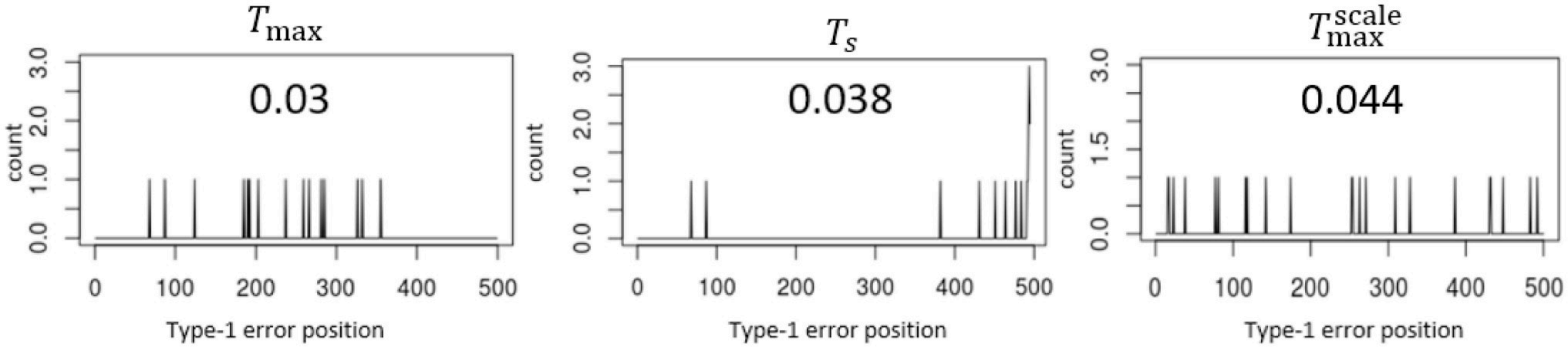
Time positions of type-1 error in Lorenz system.

Next, we evaluate the performance of the three tests: $T_{s}^{\text{UCL}}$, *T*_max_, and $T_{\text{max}}^{\text{scale}}$, using the SVR-ARMA-GARCH and ARE method to fit it into the dataset and estimate the residuals. Each simulation is conducted with the samples of size 500 and the significance level *α* = 0.05. Then, under the null hypothesis, the ratios of rejection of the three tests appear to be 0.03, 0.038, and 0.044, respectively, which are all close to *α* = 0.05. In Fig 9, the x-axis represents the time points where the type 1 error occurs, and the y-axis represents the number of occurrences in five hundred experiments.

**Fig 10:**
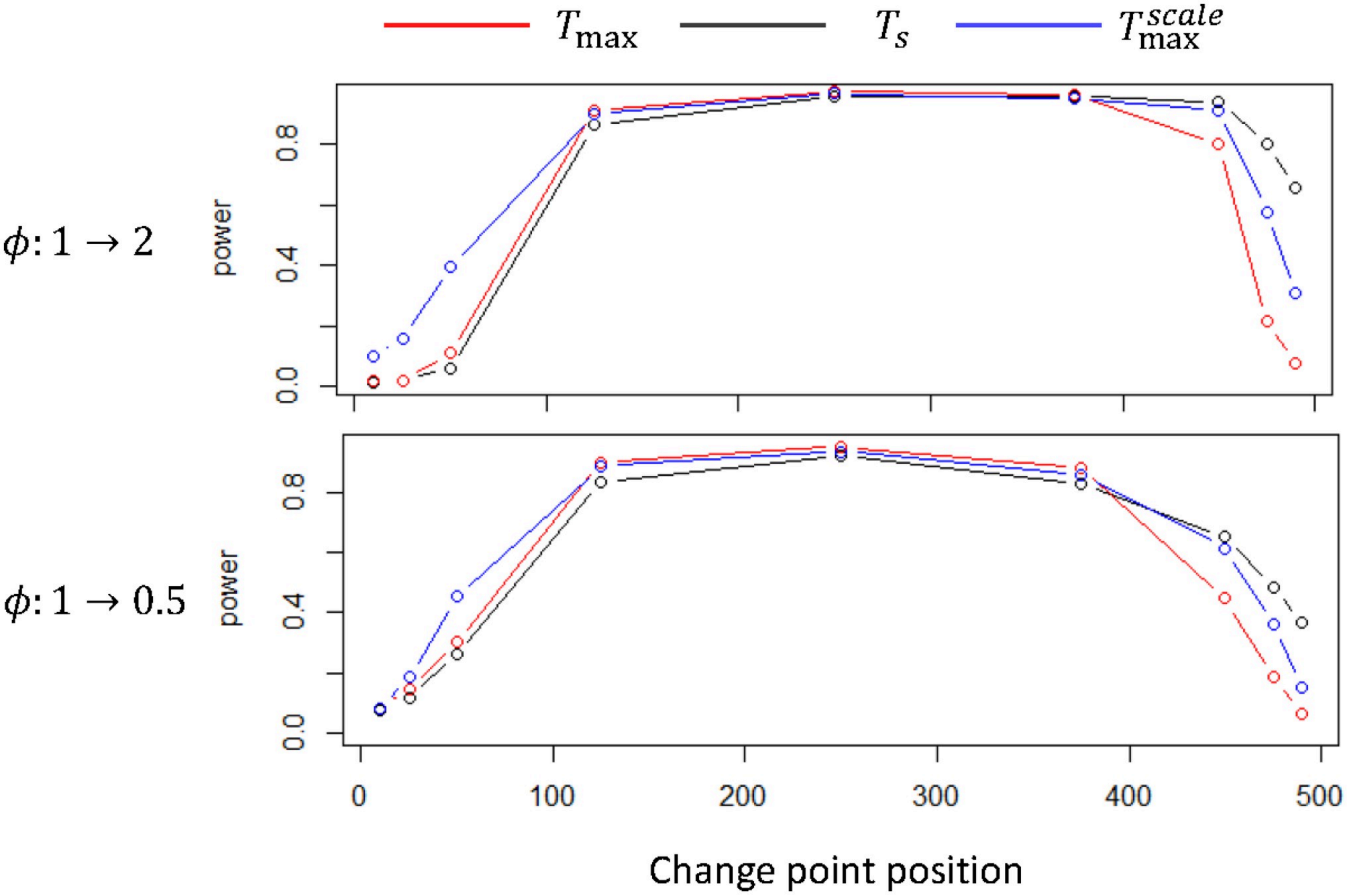
Powers in Lorenz system.

Finally, we investigate the power of the three tests by changing the value of the parameter *ϕ* in the test set at different time points and compute the proportion of rejecting the null hypothesis. Fig 10 presents the powers of the three tests at different locations. The left side presents the changed parameter. The x-axis stands for the position where the change point occurs, while the y-axis presents the rejection rate of the null hypothesis. The results exhibit that when the change point appears in an early period, the power of $T_{\text{max}}^{\text{scale}}$ is 20% higher than that of *T*_max_, and in contrast, when the change point appears in a later stage, the power of $T_{s}^{\text{UCL}}$ is 60% higher than that of *T*_max_.

### Empirical study of the BDI data

#### Data description

**Fig 11:**
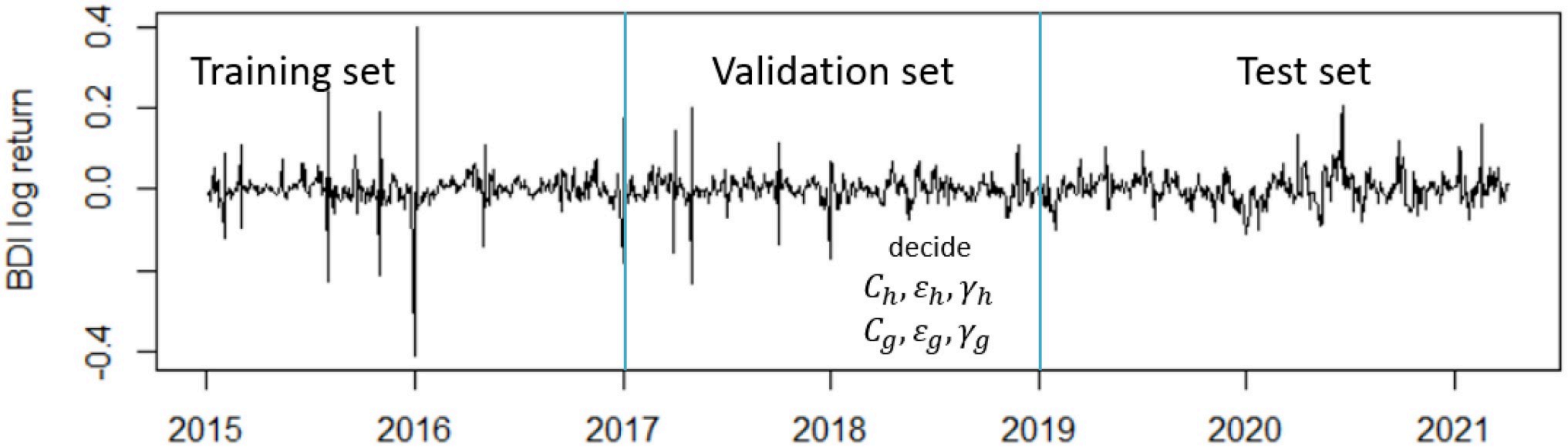
Log return of BDI.

The Baltic Dry Index (BDI) is issued daily by the Baltic Exchange in London. The BDI is a composite of the Capesize, Panamax, and Supramax Timecharter Averages. This is reported worldwide as a proxy for dry bulk shipping stocks and a general shipping market bellwether. We use the BDI daily log return data to perform a one-step-ahead forecast. We split the data into the training set from 2015/01/01 to 2016/12/31 and the test set from 2019/01/01 to 2021/04/06 concurrent with the validation set from 2017/01/01 to 2018/12/31. See Fig 11.

#### Prediction model

First, we determine the free parameters in the SVR-ARMA-GARCH model. The orders of the ARMA model and GARCH model are determined by extended autocorrelation function (EACF) and AIC. The free parameters of the SVR model are determined by a grid search. As a result, we first obtain the ARMA(2,2) model:

$$\begin{array}{c} y_{t}=0.0005+0.47y_{t-1}+0.21y_{t-2}-0.54a_{t-1}-0.02a_{t-2}+a_{t} \end{array}$$ (25)

and then an ARMA(2,2)-GARCH(1,1) model that minimizes AIC:

$$\begin{array}{c} \begin{array}{cc} y_{t} & =0.005+1.51y_{t-1}-0.49_{t-2}-1.01a_{t-1}+0.04a_{t-2}+a_{t}\\ a_{t} & =\sigma_{t}\epsilon_{t},\;\epsilon_{t}∼\left(0,1\right),\;\sigma_{t}^{2}=\text{Var}\left(a_{t}|F_{t-1}\right)\\ \sigma_{t}^{2} & =0.0003+0.633a_{t-1}^{2}+0.22\sigma_{t-1}^{2}. \end{array} \end{array}$$ (26)

We then apply a grid search to the validation set to determine the free parameters of SVR. Parameters *γ*_*h*_, *γ*_*g*_, and *ϵ*_*g*_ are selected from {0.01, 0.02, …, 0.1}, and parameter *ϵ*_*h*_ is selected from {0.1, 0.2, …, 1}. Also, parameters *C*_*h*_ and *C*_*g*_ are selected from {0.5, 0.6, …, 1.5}.

For comparison, we also consider three neural network methods: RNN, LSTM, and GRU, with two hidden layers. Here, the number of hidden layer neurons in the network is selected by a grid search from {2, 4, 8, …, 256}. We also build a neural network model for the ${\hat{a}}_{t}^{2}$ sequence to predict the conditional variance. For measuring the accuracy of prediction, we use metrics: NMES, Sign error, and RMSE_var, wherein the number of repetition is 100. Table 2 shows that the neural network model has better predictive ability than the traditional time series models (ARMA and ARMA-GARCH), but underperforms the SVR-ARMA-GARCH model, and further, the ARE method performs better in the SVR-ARMA-GARCH model.

**Table 2:** Forecast performance of six models for the BDI data.

	Traditional model		SVR-ARMA-GARCH		Neural Network (SD)		
	ARMA	ARMA-GARCH	Chen et al. [1]	ARE	RNN	LSTM	GRU
NMSE(%)	77.68	63.88	51.23	49.13	59.22(2.47)	57.31(4.12)	57.11(3.77)
Sign error(%)	34.42	28.25	17.09	16.35	22.97(1.42)	23.75(3.24)	23.30(2.39)
RMSE_var(%)	3.2498	0.2163	0.1540	0.1486	0.1956(0.018)	0.2488(0.034)	0.2128(0.011)

#### Change point detection

**Fig 12:**
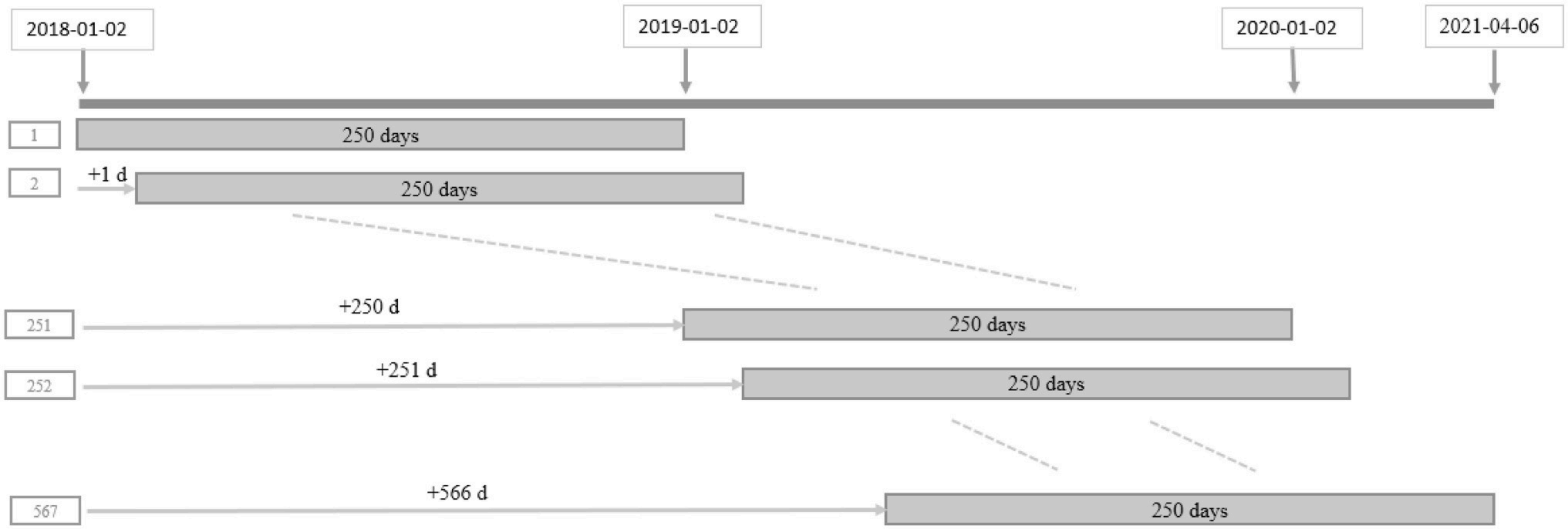
Schematic diagram of rolling window.

To perform a residual-based CUSUM test, we use the residuals obtained based on the ARE method and apply $T_{s}^{\text{UCL}}$, *T*_max_, and $T_{\text{max}}^{\text{scale}}$ to the BDI data to detect a change point from 2019/01/01 to 2021/04/06. In this task, we use the rolling window scheme with the width of one year and one day movement. See Fig 12. When a change point is detected, the point exceeding the control limit the most is declared as a change point.

**Fig 13:**
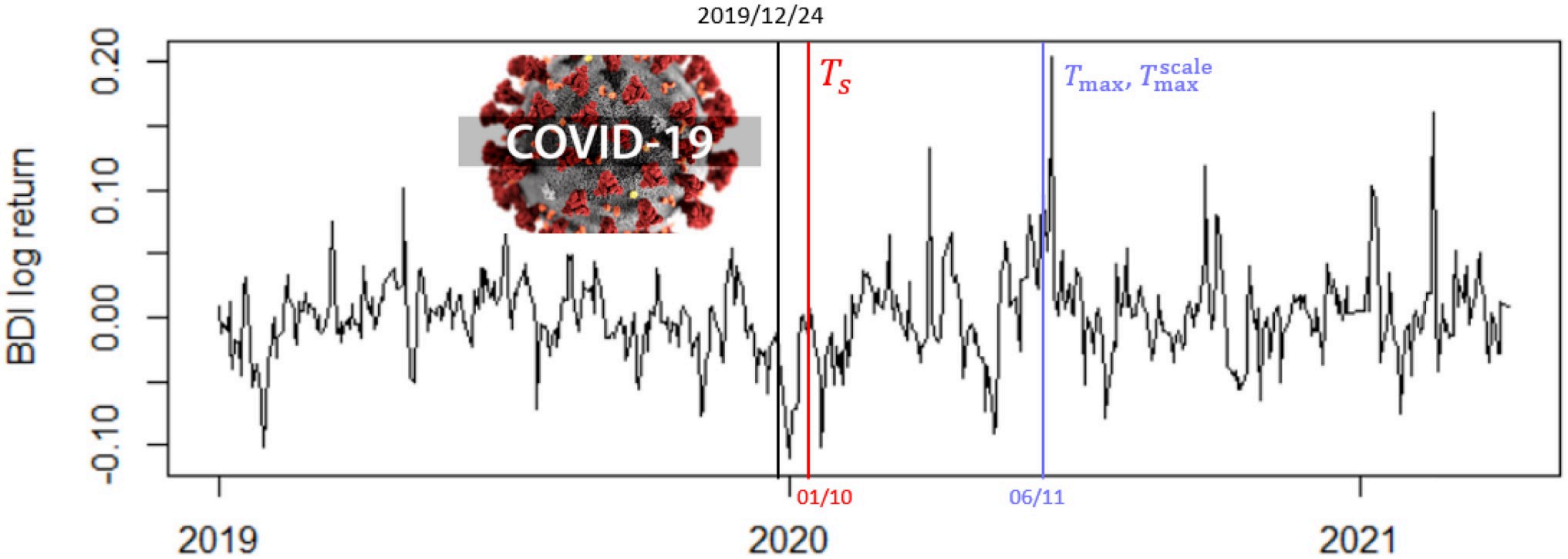
Change point of BDI: COVID-19 outbreak.

One can guess that a true change point occurs on 2019/12/24 as the COVID-19 outbreak began at that time and triggered a sudden change in the global financial market resulting in a plunge of crash in stock prices. To examine this, the three tests are conducted. The result shows that $T_{s}^{\text{UCL}}$ detects a change point most quickly on 2020/01/10, while both *T*_max_ and $T_{\text{max}}^{\text{scale}}$ detect the change point on 2020/06/11, which is quite late compared to the first test. See Fig 13.

**Fig 14:**
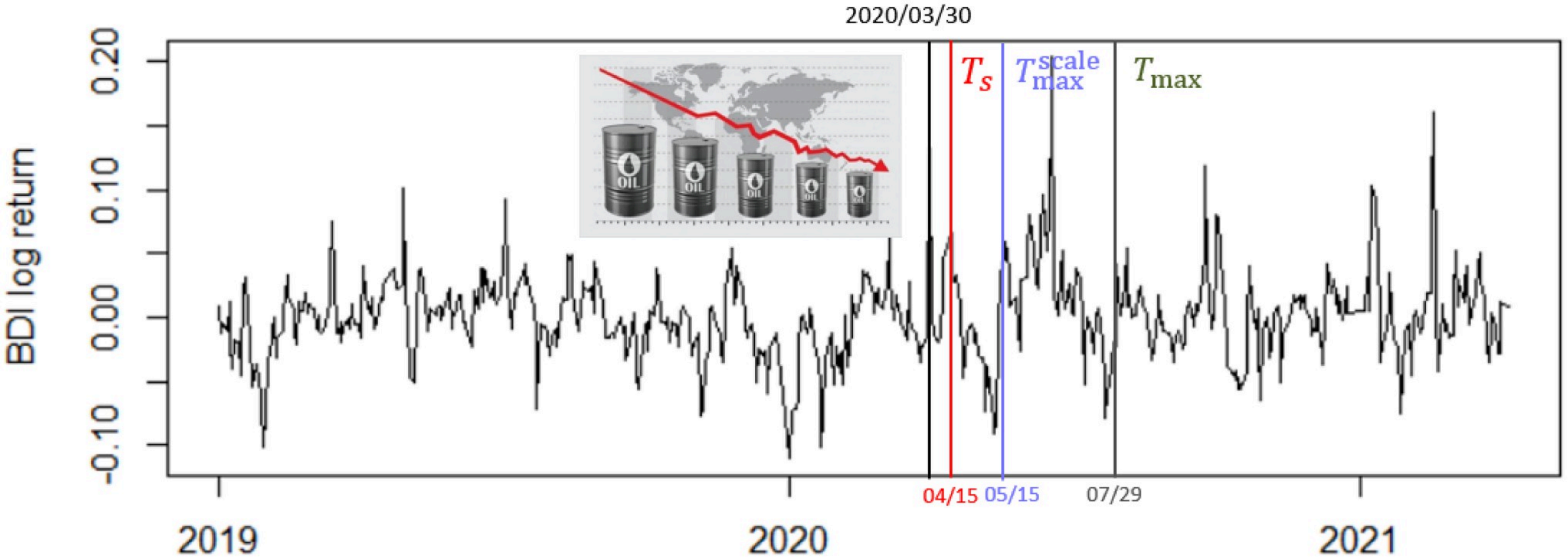
Change point of BDI: Brent crude oil fell 9%.

The second change point is claimed to be on 2020/03/30 as the price of Brent crude oil fell 9% at the date to US $23 per barrel which was the lowest price since 2002. As all ships in BDI needed oil to travel, the abnormality of Brent crude oil led to trigger a serious BDI change. In this case, $T_{s}^{\text{UCL}}$, $T_{\text{max}}^{\text{scale}}$, and *T*_max_ appear to detect a change point on 2020/04/14, 2020/05/15, and 2020/06/11, respectively. See Fig 14.

This finding reveals that $T_{s}^{\text{UCL}}$ can detect a change about a half month after the occurrence of such big events, a lot faster than *T*_max_ and $T_{\text{max}}^{\text{scale}}$, which strongly affirms the validity of our proposed method when used in tandem with the rolling window scheme.

## Conclusion

In this study, we proposed an alternating recursive estimation (ARE) for the SVR-ARMA-GARCH model. Our numerical study showed that our one-step-ahead prediction method has better predictive ability and supplies more accurate residual estimation than the method of [1]. We also proposed a new residual-based CUSUM test $T_{s}^{\text{UCL}}$ employing a time-varying control limit. We demonstrated via Monte Carlo simulations that $T_{s}^{\text{UCL}}$, *T*_max_ of [2] and $T_{\text{max}}^{\text{scale}}$ of [4] have their own advantages of producing higher powers in different time periods. Our findings in the empirical study using the daily BDI (Baltic Dry Index) data demonstrate the superiority of $T_{s}^{\text{UCL}}$ over the others in terms of quick detection ability. All these results affirmed the validity of the proposed method.

## Supporting information

S1 Data (CSV)
